# The effect of three additives on properties of mineral trioxide aggregate cements: a systematic review and meta-analysis of in vitro studies

**DOI:** 10.1186/s12903-024-04103-1

**Published:** 2024-03-14

**Authors:** Behnam Bolhari, Faranak Noori, Hadi Assadian, Amir Raee, Sholeh Ghabraei, Ahmad-Reza Shamshiri, Artak Heboyan

**Affiliations:** 1 Department of Endodontics, School of Dentistry, Tehran University of Medical Sciences (TUMS), Tehran, Iran; 2 Department of Periodontics, School of Dentistry, Tehran University of Medical Sciences (TUMS), Tehran, Iran; 3 Research Center for Caries Prevention, Dentistry Research Institute, Department of Community Oral Health, School of Dentistry, Tehran University of Medical Sciences, Tehran, Iran; 4 Department of Prosthodontics, Faculty of Stomatology, Yeravan State Medical University After Mkhitar Heratsi, 0025 Yerevan, Armenia

**Keywords:** Mineral trioxide aggregate, Calcium chloride, Propylene glycol, Compressive strength, Cell survival

## Abstract

**Background:**

Several efforts have been made to improve mechanical and biological properties of calcium silicate-based cements through changes in chemical composition of the materials. This study aimed to investigate the physical (including setting time and compressive strength) and chemical (including calcium ion release, pH level) properties as well as changes in cytotoxicity of mineral trioxide aggregate (MTA) after the addition of 3 substances including CaCl_2_, Na_2_HPO_4_, and propylene glycol (PG).

**Methods:**

The systematic review was conducted in accordance with Preferred Reporting Items for Systematic Reviews and Meta-Analyses (PRISMA).

Electronic searches were performed on PubMed, Embase, and Scopus databases, spanning from 1993 to October 2023 in addition to manual searches. Relevant laboratory studies were included. The quality of the included studies was assessed using modified ARRIVE criteria. Meta-analyses were performed by RevMan statistical software.

**Results:**

From the total of 267 studies, 24 articles were included in this review. The results of the meta-analysis indicated that addition of PG increased final setting time and Ca^2+^ ion release. Addition of Na_2_HPO_4_ did not change pH and cytotoxicity but reduced the final setting time. Incorporation of 5% CaCl_2_ reduced the setting time but did not alter the cytotoxicity of the cement. However, addition of 10% CaCl_2_ reduced cell viability, setting time, and compressive strength.

**Conclusion:**

Inclusion of 2.5% wt. Na2HPO4 and 5% CaCl2 in MTA can be advisable for enhancing the physical, chemical, and cytotoxic characteristics of the admixture. Conversely, caution is advised against incorporating elevated concentrations of PG due to its retarding effect.

**Trial registration:**

PROSPERO registration number: CRD42021253707.

## Highlights

• Total substitution of water with PG to enhance the handling properties of CSC will prevent it from complete setting.

• High concentrations of CaCl_2_ added to MTA lead to increased cytotoxicity of the admixture.

• According to conventional statistical methods, determining the sample size is of significant importance for laboratory studies to reduce the risk of bias.

## Background

Calcium silicate-based cements (CSCs) exhibit diverse applications in endodontics, encompassing procedures such as vital pulp treatments, apexification, perforation repair, and regenerative endodontic procedures [1]. ProRoot Mineral Trioxide Aggregate (ProRoot MTA; Dentsply, Tulsa, OK, USA) stands as the inaugural member of the CSCs family. Its introduction took place in 1993 comprising a composition of 75% Portland cement and 25% bismuth oxide [1]. Mineral Trioxide Aggregate (MTA) and other subsequently introduced CSCs demonstrate a diverse range of advantages, with numerous studies highlighting their notable feature of high biocompatibility [1, 2]. These materials undergo setting in the presence of moisture, yielding hydration products from the setting reaction, notably calcium silicate hydrate (CSH) gel and calcium hydroxide. The dissociation products emanating from calcium hydroxide contribute to alkalinity, and the presence of calcium ions offers an osteo/cemento inductive potential to the surrounding environment [3]. Consequently, CSCs not only demonstrate sufficient biocompatibility but also have the capability to promote the formation of a thin cementum-like layer in their vicinity. This phenomenon can be regarded as a biologic barrier, contributing to the improvement of their sealing ability [4]. In clinical applications, CSCs are positioned in direct proximity to pulpal and periodontal tissues, followed by the application of restorative materials with enhanced mechanical characteristics, such as composite resins. Consequently, while high compressive strength (CS) is not imperative for CSCs, their mechanical attributes should ensure sufficient compressive resistance against the subsequent restoration [3]. The extended setting time (ST) of CSCs, as a drawback, can impede the possibility of performing treatments in single-visit, which can therefore result in prolonged treatment time, need for scheduling further treatment sessions, and inter-appointment re-infection of the prepared root canal space [5]. According to the manufacturer's recommendation, the powder of MTA should be added to distilled water in a 3:1 ratio. This would result in a sandy and a bit gritty consistency and poor handling of the material [6].

Numerous efforts have been made to incorporate or eliminate diverse substances from the compositions of CSCs in order to enhance their properties [7]. For example, to enhance the handling characteristics, it has been suggested to add propylene glycol (PG) to the water used with MTA. Due to its higher viscosity and water solubility, a more clinically convenient consistency of MTA admixture will be provided [8]. However, the inclusion of PG can reduce the required water quantity for the setting reaction. This, in turn, minimizes the development of capillary pores within the cement structure and enhances the ultimate CS [9]. On the other hand, the presence of sufficient water is necessary to facilitate the hydration reaction of the cement. In cases of water inadequacy, the setting reaction may be interrupted, potentially compromising the CS. Collectively, increased CS of MTA following addition of PG remains to be a matter of debate and determining the proper PG ratio to incorporate in MTA composition remains to be elucidated [10, 11].

Calcium chloride (CaCl_2_) is an inorganic compound that can be created by neutralizing hydrochloric acid with calcium hydroxide. It has been demonstrated that addition of this material to Portland cement can accelerate ST by facilitating the hydration reaction [12]. Conversly, it can also reduce the amount of water required for clinical applications. By the same token, inclusion of CaCl_2_ can enhance CS of the cement via reducing its porosity [9]. This substance has been incorporated into MTA in varying proportions, as documented in the literature [9, 13, 14]. Nevertheless, consensus on the optimal percentage for the inclusion of CaCl2 remains a matter of debate.

Na_2_HPO_4_ is an inorganic compound with a pH of 9.5. This liquid can accelerate the ST of MTA by phosphate effect in cements with α-tricalcium phosphate as the powder [15]. Although several solutions and powders can provide phosphate, this formula has been suggested because of providing a more physiologic pH compared to other materials [15]. It has also been indicated that alkalinity of the environment can enhance the CS of MTA [16]. It should be noticed that any alteraltion in the setting process of CSCs can influence hydration products, which, in turn, significantly influence their biological effects.

Various studies have sporadically evaluated the effect of these substances on the mentioned properties of MTA, indicating contradictory results. Even in cases where the results of the studies were consistent, it was not possible to decide on a more appropriate concentration of the additive because heterogenous percentages of the material were examined in studies. In addition, due to a large number of studies on variable MTA properties, it was not possible to reach consensus about the effect of an additive on the most critical properties of MTA. Nevertheless, the incorporation of any substance into MTA may enhance certain properties while compromising others. To the best of the authors' knowledge, there is no comprehensive information about the effect of these additives on the critical properties of MTA. Therefore, the purpose of this systematic review was to investigate the effect of substances added to MTA on its physical, chemical, and cytotoxic properties. In case there was a consensus on the effectiveness of a certain additive on the cement in the literature, attempts have been made to provide a comprehensively reviewed information.

## Methods

The present systematic review adheres to the guidelines outlined in the Preferred Reporting Items for Systematic Reviews and Meta-Analyses (PRISMA) statement [17]. Additionally, it has been registered in the International Prospective Register for Systematic Reviews (PROSPERO) under the registration number CRD42021253707.

### Research question

The following research question was developed: Do various additives incorporated into different white MTA brands (including white ProRoot MTA, white MTA-Angelus, and white Root MTA) affect physical (including setting time and CS), chemical (including calcium ion release, pH level) properties, and cell cytotoxicity via MTT/MTS^1^ assays? This question was formulated as follows:

- Population: various MTA brands
- Intervention: incorporation of CaCl2, Na2HPO4 and PG to MTA
- Comparison: unchanged MTA formulations
- Outcome: physical, chemical, and cytotoxic properties
- Study design: in vitro studies

### Search strategy and inclusion criteria

The electronic search was conducted across three databases, namely PubMed, Embase, and Scopus, without imposing any language restrictions, spanning the period from 1993 (commencing with the introduction of the first CSC) to October 2023. The keywords of the search strategy are listed in Table 1. A manual search was performed through the articles, conference abstracts, and letters published in two leading endodontic journals, namely "*Journal of Endodontics"* and *"International Endodontic Journal"* regarding the search question during the last three years.

**Table 1:** Electronic databases and research strategies

Pubmed
((((((((((MTA[MeSH Terms]) OR (MTA bio[Title/Abstract])) OR (mineral trioxide aggregate[Title/Abstract])) OR (MTA-Angelus[Title/Abstract])) OR (portland cement[Title/Abstract])) OR (calcium silicate cement[Title/Abstract]))) OR (Calcium Silicate-based Cement[Title/Abstract])) OR (accelerated portland cement[Title/Abstract])) AND (((((propylene glycol[Title/Abstract]) OR (disodium hydrogen phosphate[Title/Abstract])) OR (Na2HPO4[Title/Abstract])) OR (calcium chloride[Title/Abstract])) OR (CaCl_2_ [Title/Abstract]))) AND (((((((((setting time[Title/Abstract])) OR (compressive strength[Title/Abstract])) OR (calcium ion release[Title/Abstract])) OR (pH[Title/Abstract])) OR (pH value[Title/Abstract])) OR (biocompatibility[Title/Abstract])) OR (cytotoxicity[Title/Abstract])) OR (cell viability[Title/Abstract]))
Embase
mta:ab,ti OR 'mta bio':ab,ti OR 'mineral trioxide aggregate':ab,ti OR 'mta angelus':ab,ti OR 'portland cement':ab,ti OR 'calcium silicate cement':ab,ti OR 'calcium silicate-based cement':ab,ti OR 'accelerated portland cement':ab,ti AND 'propylene glycol':ab,ti OR 'disodium hydrogen phosphate':ab,ti OR na2hpo4:ab,ti OR 'calcium chloride':ab,ti AND 'setting time':ab,ti OR 'compressive strength':ab,ti OR 'calcium ion release':ab,ti OR ph:ab,ti OR biocompatibility:ab,ti OR cytotoxicity:ab,ti OR 'cell viability':ab,ti
Scopus
( ( TITLE-ABS-KEY ( mta) OR TITLE-ABS-KEY ( "MTA bio") OR TITLE-ABS-KEY ( "mineral trioxide aggregate") OR TITLE-ABS-KEY ( "MTA-Angelus") OR TITLE-ABS-KEY ( "portland cement") OR TITLE-ABS-KEY ( "calcium silicate cement") OR TITLE-ABS-KEY ( "Calcium Silicate-based Cement") OR TITLE-ABS-KEY ( "accelerated portland cement"))) AND ( ( TITLE-ABS-KEY ( "propylene glycol") OR TITLE-ABS-KEY ( "disodium hydrogen phosphate") OR TITLE-ABS-KEY ( na2hpo4) OR TITLE-ABS-KEY ( "calcium chloride") OR TITLE-ABS-KEY (CaCl_2_))) AND ( ( TITLE-ABS-KEY ( "setting time") OR TITLE-ABS-KEY ( "compressive strength") OR TITLE-ABS-KEY ( "calcium ion release") OR TITLE-ABS-KEY ( ph) OR TITLE-ABS-KEY ( biocompatibility) OR TITLE-ABS-KEY ( cytotoxicity) OR TITLE-ABS-KEY ( "cell viability")))

The search strategy used for each database was defined in Table 1. The references of the included studies (cross-referencing) and published systematic reviews were also searched for potentially relevant articles.

### Eligibility criteria

Laboratory studies that evaluated the effect of adding different substances(including CaCl_2_, Na_2_HPO_4_, and PG) to commercially available white MTA cements on the physical (including setting time and CS), chemical (including calcium ion release, pH level) and biological properties (cell cytotoxicity via MTT/MTS assays) of the material were included. The study excluded research lacking a control group, investigations solely focusing on the physical properties and alterations of the substance in diverse environments, inquiries into distinct techniques and additives related to non-dental Portland cement formulations, examinations of MTA-like experimental cements as opposed to the introduction of diverse substances into commercially available CSCs, animal studies, clinical trials, case reports, case series, editorials, expert opinions, letters, reviews, and conference abstracts. To maintain study homogeneity, commercialy available white MTAs with relatively similar powder compositions (such as ProRoot MTA, MTA-Angelus, Ortho MTA, and Root MTA) were included.

### Screening and selection

The studies were screened independently by two researchers (B.B and F.N) to identify those with titles and abstracts meeting the inclusion criteria. The articles that received consensus from both authors were chosen, and any disagreements were resolved through the intervention of a third reviewer (S.G). The full-text versions of studies meeting the inclusion criteria were obtained, and a quality assessment was conducted using modified ARRIVE (Animal Research: Reporting of In Vivo Experiments) criteria [18].

### Data extraction

A data extraction table was formulated, encompassing the following particulars acquired from the chosen studies: author's name, year of publication, PICOS-related items (population, intervention, comparison, outcome, and study design), sample size, time interval, and methodology. When deemed essential, supplementary data were acquired by reaching out to the study authors via email.

### Quality assessment

Two reviewers (B.B., F.N.) independently assessed the quality of included studies. For each study, risk of bias assessment was evaluated based on a previous investigation (modified ARRIVE) [18] and verified whether the physical, chemical, and cytotoxic properties were analyzed in accordance with the following parameters: (a) standardization of the procedures for preparing the samples, (b) single operator, (c) description of sample size calculation, (d) blinding of the test machine operator, and (e) conduction of the tests in accordance with standard specifications.

In case the article thoroughly assessed the parameter, a score of 0 was assigned for that specific parameter. If the article reported the particular parameter but provided an inadequate or unclear description, it received a score of 1. If no information was available on the parameter of interest, the article was given a score of 2. Scores were cumulated across the five parameters. Articles that received a total score between 0 and 3 were categorized as having a low risk of bias, those scoring 4 to 7 were classified as moderate, and those with scores between 8 to 10 were considered to have a high risk of bias.

Any disagreement was resolved through discussion with a third reviewer (S.G.) to achieve a consensus.

### Data synthesis

To perform a meta-analysis, RevMan statistical software (Revman version 5.4; Cochrane, London, UK) was used to analyze the same outcome measures of comparable studies. Since the extracted data was continuous, a weighted mean difference (WMD) with a 95% confidence interval (CI) was used for reporting the data. Statistical heterogeneity was assessed using Cochrane's Q-statistics. The heterogeneity among studies was also quantified using I^2^ statistical test [19], and I^2^ > 50% was considered as a statistically significant heterogeneity. In case of heterogeneity, a random effects model was applied to pool the results. Otherwise, a fixed-effect model was used if no heterogeneity was observed.

Because less than 10 studies were included for each meta-analysis, the publication bias was not assessed due to the limited power to detect publication bias [20]. Although the data included in a single meta-analysis may have been more than ten items, this data was extracted from less than ten articles.

## Results

### Search and selection

**Fig. 1:**
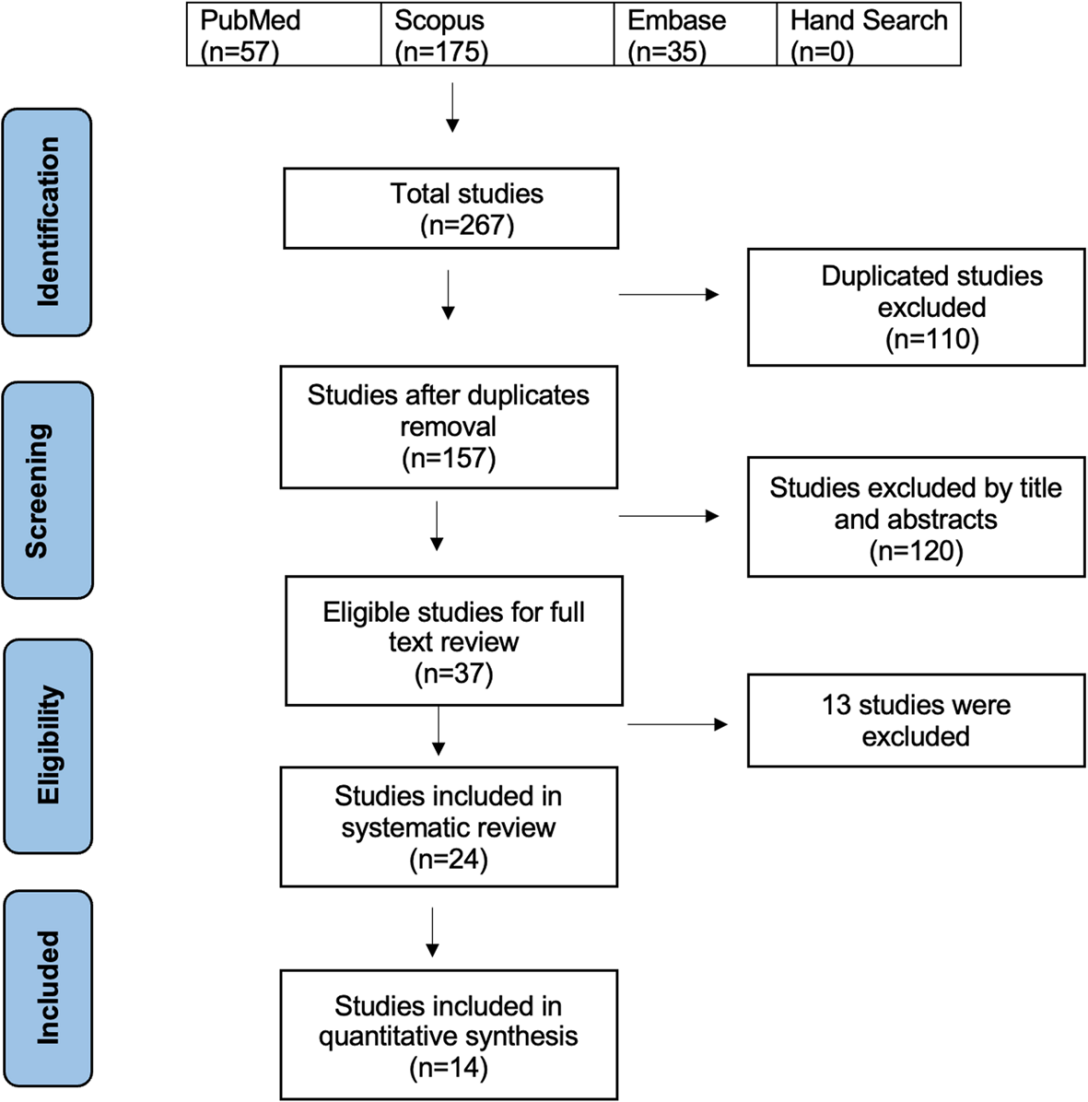
The PRISMA flow diagram

The flow diagram of the selection process is drawn in Fig. 1. A total of 267 studies were identified through the electronic search, and the first one was published in 2006. Among all, 110 duplicates were excluded. After screening titles and abstracts, 120 articles were excluded because of irrelevance. The remaining 37 studies were selected for appraisal of the full-text version. Eleven articles were excluded, because they did not meet the inclusion criteria (i.e. use of animal models, evaluating multiple additives in one sample, not using commercially available CSC). |One study was excluded because of not reporting the percentage of the additive material [21]. One other study was excluded because PG was extracted from a natural source, which could make differences from commercially available ones [22]. Finally, 24 articles were included for the systematic review. The main characteristics of the included articles are summarized in Table 2.

**Table 2:** Main characteristics of the included studies

Author, year	Additive			Property					n	Results [± SD]	Conclusion
	CaCl_2_	Na2HPO4	PG^a^	ST^b^	CS^c^	pH	Ca^2+^ ion release	cytotoxicity			
Jamali Zavare, F. 2020 [23]	5% CaCl_2_						X		3	MTA 1 day: 281 ± 8 Day 7:318 ± 15 Day 14: 761 ± 34 MTA + 5% CaCl_2_ 1 day: 268 ± 16 Day 7: 607 ± 8 Day 14: 810 ± 15	The addition of CaCl_2_ to MTA and CEM cement decreased their setting time and increased pH and Ca^2+^ ion release
Mokhtari, H. 2018 [24]		2.5% wt. Na2HPO4			X				10	1D WMTA + liquid 50%: 42.15 ± 1.50 60%: 41.33 ± 2.09 70%: 37.79 ± 1.28 Na_2_HPO_4_ WMTA + liquid 50%: 41.44 ± 1.77 60%: 43.72 ± 1,78 70%: 37.37 ± 1.62 21 D WMTA + liquid 50%: 63.25 ± 1.96 60%: 59.51 ± 1.50 70%: 57.27 ± 1.26 Na_2_HPO_4_ WMTA + liquid 50%: 62.39 ± 1.39 60%: 63.96 ± 1.40 70%: 59.78 ± 1.03	Adding 2.5% wt. Na_2_HPO_4_ to MTA increased samples CS
Mokhtari, H. 2018 [25]		2.5% wt. Na2HPO4		X					30	Final setting time WMTA = 182.00 ± 57.86 NAMTA = 67.00 ± 14.42	Adding 2.5% wt. Na_2_HPO_4_ to MTA reduces ST
Kulan, P. 2018 [26]	5% CaCl_2_	2.5% Na2HPO4						MTS	3	CaCl_2_ 5% 1 d = 100 ± 10 7 d = 74.4 ± 14 21 d = 81.3 ± 14 Na_2_HPO_4_ 2.5% 1 d = 91.4 ± 31 7 d = 56.1 ± 10 21 = 40.9 ± 15 DW 1 d = 97.8 ± 2.1 7 d = 73.4 ± 1.1 21 d = 38.7 ± 20	All MTA samples increased the proliferation of DPSCs
Ahmed, H. 2018 [14]	10% CaCl_2_							MTS	3	24h 200 mg/ml (full concentration) DW: 21.8 ± 14.5 Fs: 0 72 h 200 mg/ml DW: 10.1 ± 1.4 Fs: 0	addition of CaCl_2_.2H_2_O increases the cytotoxicity but enhances the dentinogenic differentiation potential of MTA on DPSCs
Ahmad, A. 2017 [27]	10% CaCl_2_							MTT	3	24h 50mg/ml Fs = 10 ± 5 DW = 17.5 ± 2.5 72h 50mg/ml Fs = 2.5 ± 2 DW = 20 ± 7.5 DW = 112 ± 22	Admixture of 10% CaCl_2_ with MTA has a favorable biological profile towards HPLFs
Sobhnamayan, F. 2017 [28]			20%, 50% and 100% PG		X				15	mean (median) ± SD DAY 7 100% PG: 20 (20) ± 4.56 50% PG: 22 (22.3) ± 2.52 20% PG: 18 (19.9) ± 6.31 100% DW: 10 (11) ± 3.20	The appropriate concentration of PG could improve the CS of MTA and CEM cement
Marciano, M. A. 2016 [6]			20% PG	X		X	X		10	MTA + DW Initial setting time (min): 13.60 ± 1.30 Final setting time: 68.33 ± 1.53 MTA + PG Initial setting time 17.31 ± 1.40 Final setting time 103.00 ± 3.35	The addition of PG to MTA resulted in a longer final setting time than MTA + DW For MTA + PG, higher values of pH and Ca release were observed in the final period of 168 h
Kulan, P. 2016 [29]	5%, 10% CaCl_2_,	2/5% wt. Na2HPO4						MTS	3	CaCl_2_ 10% 1D = 51.6 ± 18.6 3 d = 50.6 ± 8 7 d = 28.5 ± 8 CaCl_2_ 5% 1 d = 64.1 ± 9 3 d = 70.1 ± 9.8 7 d = 57.1 ± 4.2 Na_2_HPO_4_ 2.5% 1 d = 45.3 ± 15 3 d = 54.9 ± 8 7 d = 56.4 ± 3 DW 1 d = 67.2 ± 9 3 d = 70.4 ± 9.8 7 d = 57.1 ± 4.2	The samples of MTA mixed with 5% CaCl_2_ and Na_2_HPO_4_ were statistically more biocompatible than the samples of MTA mixed with 10% CaCl_2_
Ghasemi, N. 2016 [10]			20% PG		X				*N* = 15	Mean (SD) MTA + DW 4 days: 35.85 (12.34) 21 days: 51.22 (18.92) MTA + PG 4 days: 4.5 (0.67) 21 days: 16 (6.78)	Addition of 20% PG reduces CS of MTA
Zapf, A. M. 2015 [30]	5% CaCl_2_	15% Na2HPO4		Ca(OH)^2^ decomposition enthalpy					3		CaCl_2_ accelerated the reaction product formation
Prasad 2015 [13]	10% CaCl_2_,			X	X	X			10	Final setting time MTA + DW = 133.10 ± 7.84 MTA + 10% CaCl_2 =_ 25.40 ± 5.58 MTA + 15% Na_2_HPO_4_ = 31.06 ± 2.91 pH 24h MTA + DW = 12.54 ± 0.27 MTA + 10% CaCl_2 =_ 11.22 ± 0.15 MTA + 15% Na_2_HPO_4_ = 12.77 ± 0.09 CS 1day MTA + DW = 18.40 ± 0.64 MTA + 10% CaCl_2 = 10.82_ ± 1.08 _MTA_ + 15% Na_2_HPO_4_ = 12.76 ± 1.60 3day MTA + DW = 23.74 ± 1.25 MTA + 10% CaCl_2_ = 18.72 ± 0.65 _MTA_ + 15% Na_2_HPO_4_ = 24.37 ± 1.06 7day MTA + DW = 36.24 ± 3.33 MTA + 10% CaCl_2_ = 33.37 ± 3.18 _MTA_ + 15% Na_2_HPO_4_ = 29.32 ± 1.13	10% CaCl_2_ and 15% Na_2_HPO_4_ significantly reduced the setting time of MTA By adding 10% CaCl_2_ and 15% Na_2_HPO_4_ the pH maintained at a high value There was no improvement in the CS of the material
Natu, V. P. 2015 [11]			20%, 50% and 100% PG	X		X	X		5 3 2	W/PG (initial setting time) 100/0 = 18.3 ± 0.3 80/20 = 55.9 ± 0.7 50/50 = 191.0 ± 0.5 pH and Ca^2+^ ion release The numbers are not reported	addition of PG did not improve the chemical and physical properties of MTA
Lee, B. N. 2014 [31]	10% CaCl_2_							MTS	10	48h Relative cell viability DW: 114 ± 5 Fs: 115 ± 4	There was no significant difference in cell viability between experimental groups
Oloomi, K. 2013 [32]	5% CaCl_2_	2.5% wt. Na2HPO4			X				5	1h DW: not set CaCl_2_: 15.64 ± 2.05 Na2HPO4: 19.66 ± 1.25 3h (sig) DW: 17.36 ± 3.11 CaCl_2_: 41.20 ± 7.08 Na2HPO4: 38.16 ± 3.85 24h DW: 44.52 ± 3.52 CaCl_2_: 48.02 ± 2.93 Na2HPO4: 46.26 ± 3.56 1week DW: 62.64 ± 3.28 CaCl_2_: 60.08 ± 3.60 Na2HPO4: 58.64 ± 5.42	CS of original and accelerated RMTA was not significantly different after one week
Kang, J. Y 2013 [33]	10% CaCl_2_						X	XTT	6	Ca^2+^ ion release in 1day MTA = 3.781 mg/dl MTA + 10% CaCl_2_ = 331.1 XTT 1D Fs = 70 ± 8.3 DW = 82.5 ± 6.3 4D Fs = 60 ± 8.3 DW = 85.5 ± 8.3 7D Fs = 73 ± 18.7 DW = 93 ± 5.2	MTA mixed with 10% CaCl_2_ in all groups showed the lowest cell viability at every time point and released a higher amount of Ca^2+^ ions than the other groups
Duarte, M. A. 2012 [8]			20%, 50%, 80% and 100% PG	X		X	X		10	Initial setting time 100% DW = 15 ± 1.4 80% DW 20% = 45 ± 1.8 50% DW 50% PG = 175 ± 1.8 20% DW 80% PG = 403 ± 24.6 100% PG = not set Final setting time 100% DW = 30 ± 1.6 80% DW 20% = 85 ± 2.2 50% DW 50% PG = 385 ± 8.4 20% DW 80% PG = 661 ± 10.2 100% PG = not set pH 100% DW = 7.80 ± 0.37 80% DW 20% = 7.44 ± 0.19 50% DW 50% PG = 7.56 ± 0.05 20% DW 80% PG = 7.61 ± 0.22 100% PG = 7.61 ± 0.27 Control = 6.90 ± 0.25 Ca^2+^ ion release Mg/l 24h 100% DW = 3.10 ± 0.78 80% DW 20% = 4.89 ± 1.01 50% DW 50% PG = 4.40 ± 0.91 20% DW 80% PG = 4.36 ± 2.47 100% PG = 3.93 ± 1.49 Control = 0	The addition of PG to MTA-Angelus increased ST. Also increased the pH and Ca^2+^ ion release during the initial and post-mixing periods
Lee, B. N. 2011 [3]	10% CaCl_2_			X	X	X			10	Final setting time MTA + DW = 108.1 ± 1.6 MTA + 10% CaCl_2 =_ 74.0 ± 0.6b CS MTA + DW 1 day = 19.86 ± 3.74 3 day = 37.06 ± 5.10 7 day = 39.08 ± 3.12 MTA + 10% CaCl_2_ 1 day = 10.79 ± 1.88 3 day = 19.31 ± 1.93b 7 day = 35.30 ± 6.67 pH 24h MTA + DW = 12.9 ± 0.1 MTA + 10% CaCl_2_ = 11.5 ± 0.2	Addition of 10% CaCl_2_ to MTA decreased ST Also decreased CS at all time points for 7 days, but there was no difference in the day 7 The pH of the admixture was significantly lower than the control, but maintained in high level but stable at a high level (pH 11–12)
Jafarnia, B. 2009 [34]	5% CaCl_2_							MTT	6	Set MTA 1D DW: 91 ± 2 Saline: 89 ± 3 Fs: 90 ± 4 2D DW: 85 ± 4 Saline: 83 ± 5 Fs: 81 ± 7 3D DW: 83 ± 3 Saline: 81 ± 3 Fs: 84 ± 2	The addition of 5% CaCl_2_ does not affect the cytotoxicity of MTA
Bortoluzzi, E. A. 2009 [9]	10% CaCl_2_			X		X			36	Initial setting time WMTA = 12 ± 0.34 WMTA + CaCl_2_ = 6 ± 0.50 Final setting time WMTA = 48 ± 0.87 WMTA + CaCl_2_ = 31 ± 2.00 pH Immediate DW = 9.77 ± 0.18 10% CaCl_2_ = 10.06 ± 0.13 24h DW = 11.07 ± 0.02 10% CaCl_2_ = 11.29 ± 0.09	The addition of CaCl_2_ to MTA reduced both the initial and final ST and significantly increased the pH of MTA in the immediate period, at 24 h, and at 72 h
Huang, T. H. 2008 [15]		15%, 10% and 5% Na2HPO4		X		X			3	Final setting time DW = 151 ± 6 5% = 108 ± 5 10% = 89 ± 4 15% = 26 ± 2 pH After initial mixing = 11.0 20min = 12.0 Steady point = 13.2	The Na_2_HPO_4_ solution may be an effective setting accelerator for MTA
Ding, S. J. 2008 [35]		15%, 10% and 5% Na_2_HPO_4_		X		X		XTT	3	Final setting time 5% = 124 ± 12 10% = 100 ± 14 15% = 28 ± 2 DW = 228 ± 12 pH Freshly mixed = 11.0 2 h = 12.5 6 h after final setting = 13.5 XTT 1D 15% = 89 ± 11 DW = 92 ± 11 7D 15% = 94 ± 12 DW = 100 ± 13	The ST decreased as the concentrations of Na_2_HPO_4_ increased The cell survival rate was higher than 90%
Wiltbank, K. B. 2007 [5]	5% CaCl_2_			X		X			3	Initial setting time WMTA DW = 74.4 ± 26.1 5% CaCl_2_ = 35.1 ± 7.2 GMTA DW = 67.5 ± 9.9 5% CaCl_2_ = 33.3 ± 4.5 pH	Adding 5% CaCl_2_ reduced the ST but did not change the pH significantly
Antunes Bortoluzzi, E. 2006 [36]	10% CaCl_2_					X	X		3	Ca^2+^ ion release Mg/dl immediate DW = 0.086 ± 0.04 10% CaCl_2_ = 0.13 ± 0.06 1h DW = 0.086 ± 0.05 10% CaCl_2_ = 0.13 ± 0.05 24h DW = 1.16 ± 0.76 10% CaCl_2_ = 1.85 ± 0.45 pH Immediate: DW = 9.33 ± 0.41 CaCl_2_ = 10 ± 0.10 1 h DW = 10.93 ± 0.38 CaCl_2_ = 10.73 ± 0.25 24 h DW = 11.46 ± 0.14 CaCl_2_ = 11.46 ± 0.30	The addition of CaCl_2_ to MTA significantly increased Ca2^+^ ion release but did not change the pH significantly at 24h

^a^Propylene glycol

^b^Setting time

^c^Compressive strength

### Addition of PG

#### Effect on CS

Two studies [10, 28] evaluated CS of MTA with the addition of PG in static loading conditions. One study showed that adding 100% and 50% PG significantly increased the CS of MTA compared with control after seven days. The other study showed that incorporation of 20% PG significantly reduced CS on days 4 and 21. Because CS had been evaluated at different time points giving rise to a significantly heterogeneous data, it was impossible to perform a meta-analysis.

#### Effect on ST

**Fig. 2:**
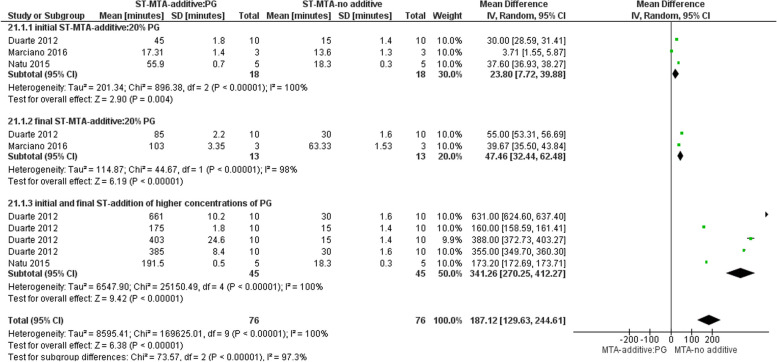
Forest plot of the meta-analysis evaluating the effect of PG addition to MTA on ST. A significant increase in the initial and final ST of MTA with the addition of 20% PG is noted. PG = propylene glycol, ST = setting time

Three studies evaluated ST of MTA following addition of PG [6, 8, 11]. The result of the meta-analysis revealed a significant increase in initial and final ST of MTA after addition of 20% PG. It was shown that addition of PG could act as a retarder for MTA setting process. Increased ST of MTA was observed with increasing the percentage of incorporated PG (Fig. 2). Accordingly, incorporation of 100% PG resulted in abortion of the setting reaction [8].

#### Effect on Ca^2+^ ion release

Three studies evaluated Ca^2+^ ion release of MTA following incorporation of different concentrations of PG using atomic absorption spectrophotometer [6, 8, 11]. In all studies, the amount of Ca^2+^ ion release after 168 h was higher in the additive group in comparison with control. Because only one study [8] reported the numeric value of Ca^2+^, it was not possible to perform a meta-analysis.

#### Effect on pH

Three studies evaluated the pH value of MTA with added different concentrations of PG [6, 8, 11].

Two studies showed that PG incorporation did not significantly change pH over longer time intervals. However, in another study, at 168 h, higher pH values were observed in MTA mixed with 20% PG [6]. A meta-analysis could not be performed because only one study [8] reported the numeric value of pH.

### Addition of disodium hydrogen phosphate (Na_2_HPO_4_)

#### Effect on CS

Three studies evaluated CS of MTA with addition of Na_2_HPO_4_ in static loading conditions [13, 24, 32]. Two out of three studies concluded that the addition of Na2HPO4 resulted in a higher CS in longer time intervals in comparison with MTA mixed with distilled water. One study showed that the CS did not alter significantly. However, because these studies used different liquid to powder ratios, different concentration of Na_2_HPO_4_, and different time points of evaluation, a meta-analysis could not be performed.

#### Effect on ST

**Fig. 3:**
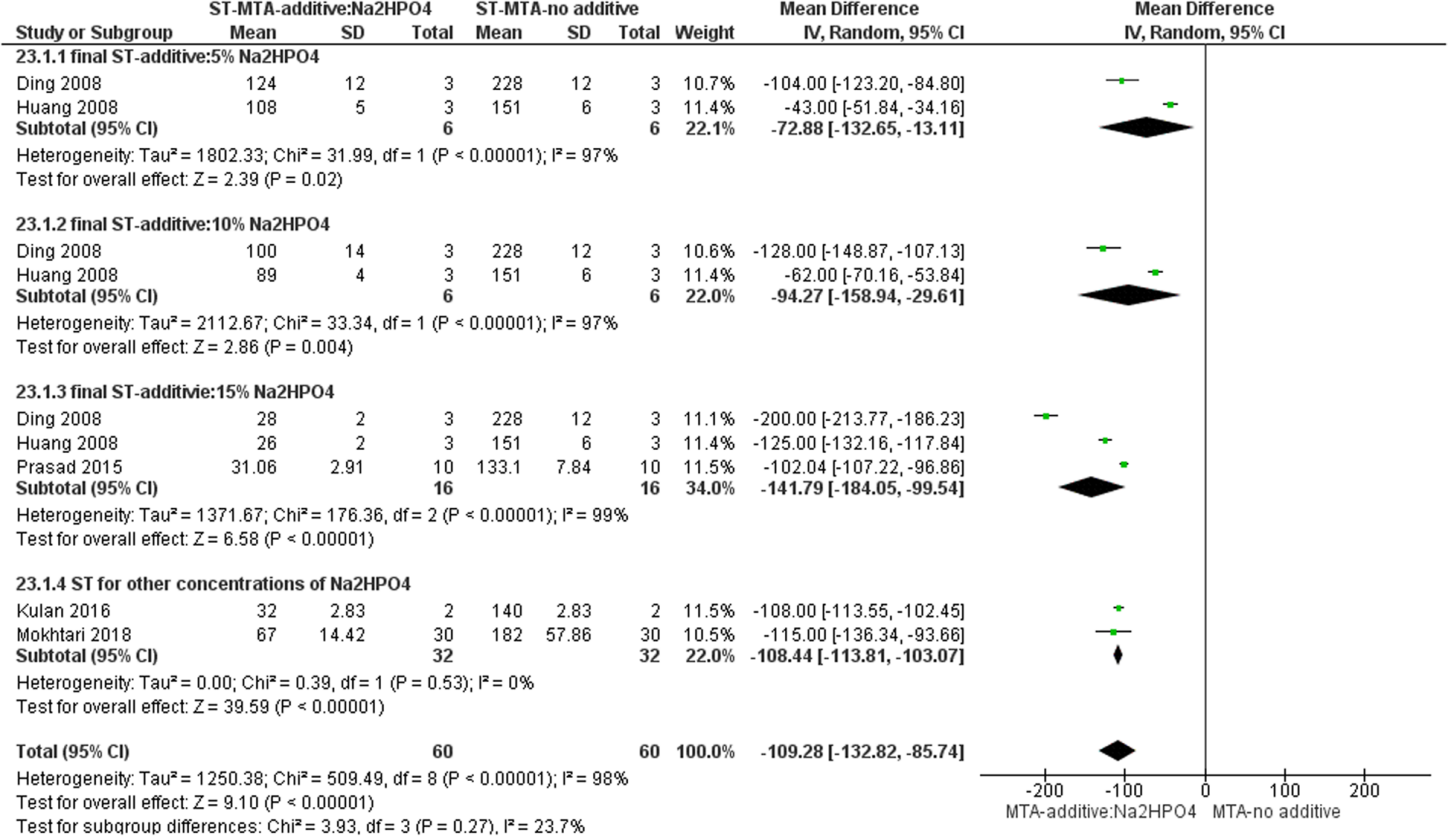
Forest plot of the meta-analysis evaluating the effect of Na2HPO4 addition to MTA on ST. The addition of Na2HPO4 decreased the final setting time, as with a higher percentage added, a more significant reduction in ST was evident. ST = setting time

Five studies evaluated the ST of MTA with the addition of different amounts of Na_2_HPO_4_ [13, 15, 25, 29, 35]. The meta-analysis result showed that adding Na_2_HPO_4_ decreased the final ST, as with a higher percentage added, a more significant reduction in ST was evident (Fig. 3). Because none of the studies evaluated the initial ST, it was impossible to perform a meta-analysis for this subject.

#### Effect on pH

Three studies evaluated the pH value of MTA with added Na_2_HPO_4_ [13, 15, 35]. They showed that the pH value for MTA mixed with distilled water and 15% Na_2_HPO_4_ did not exhibit any significant difference after the final setting of the MTA. It was not possible to perform a meta-analysis due to the lack numeric data.

#### Cytotoxicity

**Fig. 4:**
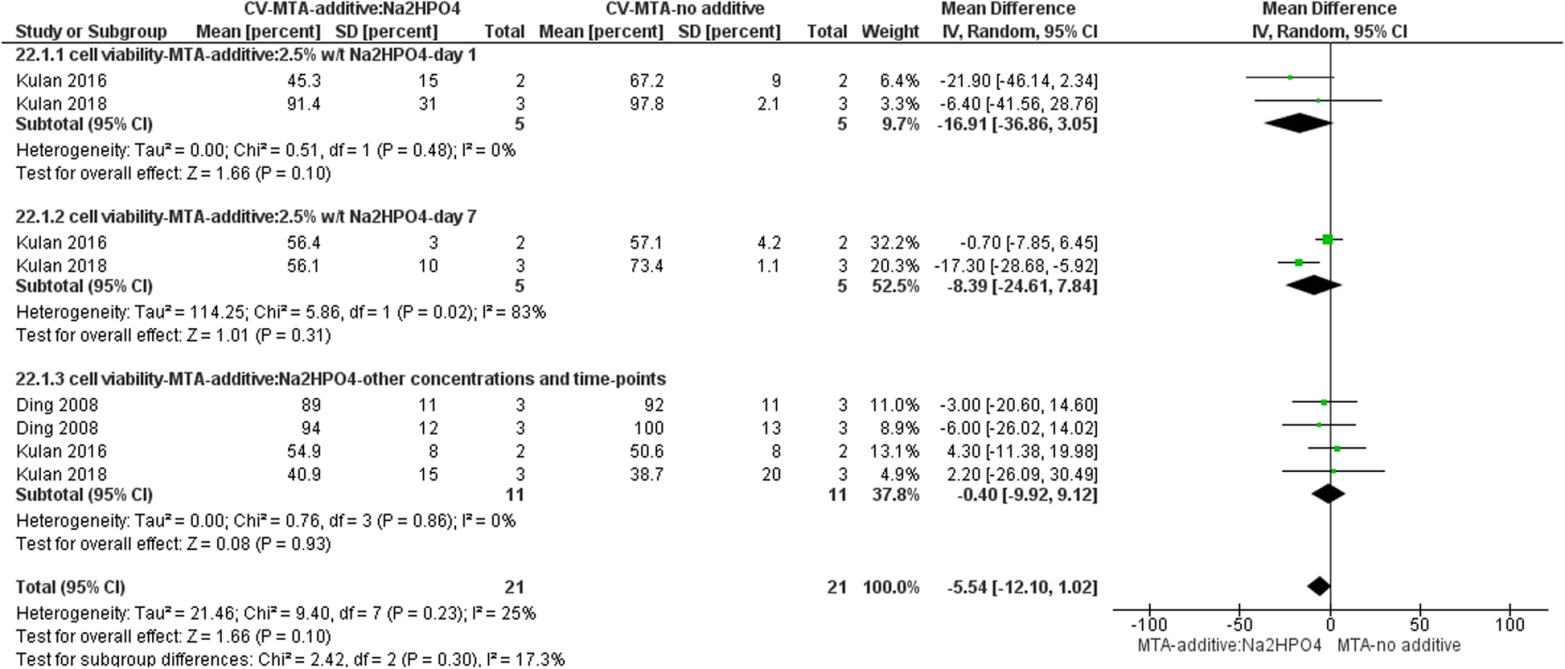
Forest plot of the meta-analysis evaluating the effect of Na2HPO4 on cytotoxicity. Regardless of additive concentration, there is no significant difference between MTA with added Na2HPO4 and MTA with no additive in 1 to 7 days. CV = cell viability

Three studies evaluated the cytotoxicity of Na_2_HPO_4_ added to MTA via MTT/MTS assays [26, 29, 35]. The meta-analysis revealed that concerning cell viability as measured by the MTT/MTS assay, there was no significant difference between MTA supplemented with Na2HPO4 and MTA without any additive within the time range of 1 to 7 days, irrespective of the concentration of the additive (Fig. 4).

### Addition of calcium chloride (CaCl_2_)

#### Effect on CS

**Fig. 5:**
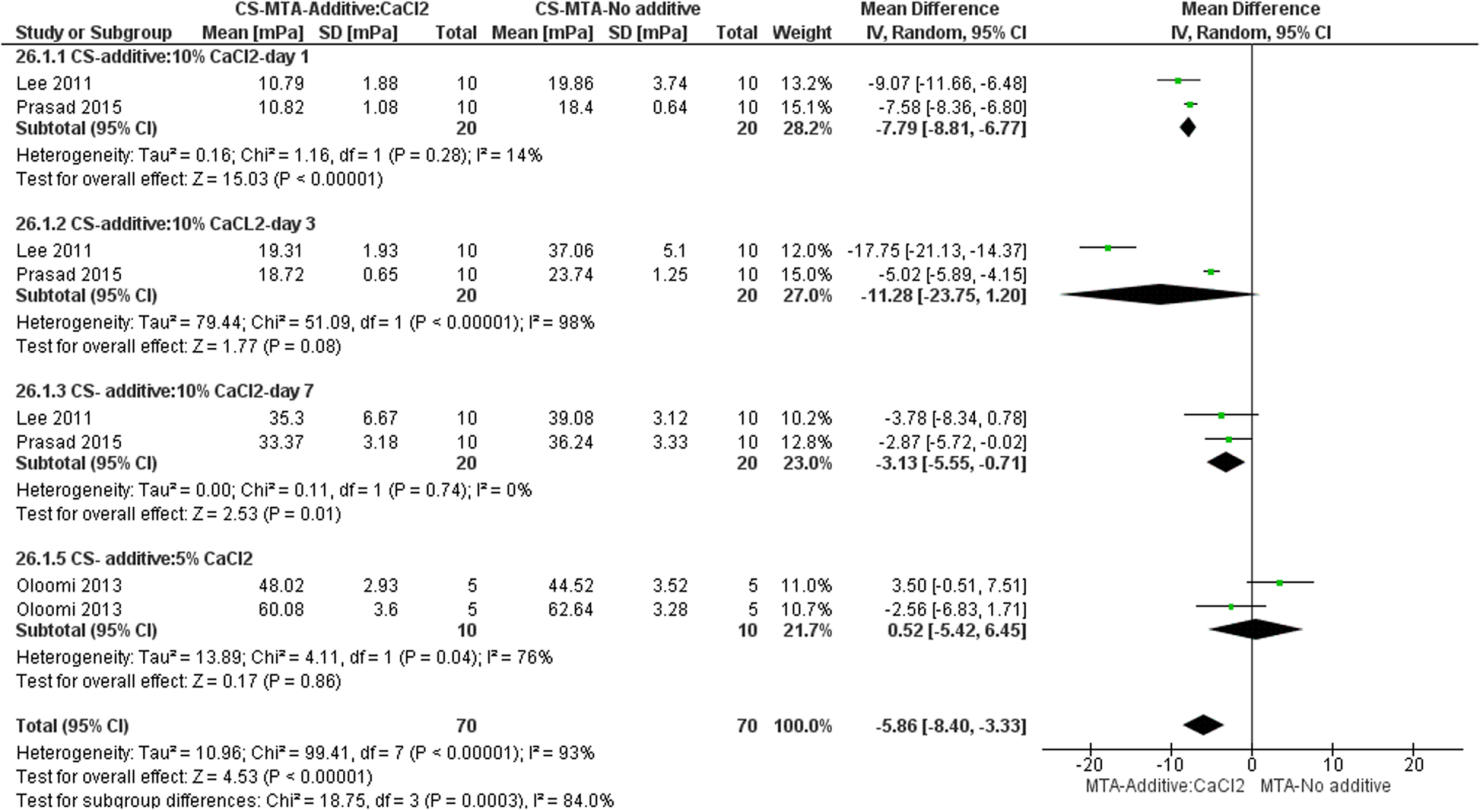
Forest plot of the meta-analysis evaluating the Effect of CaCl2 on CS. In general, regardless of time point and the percentage of the additive, CaCl2 addition resulted in a reduction of CS. However, the subgroup meta-analysis indicated that the addition of 10% CaCl2 to MTA resulted in decreased CS on days 1 and 7 compared to MTA mixed with DW. However, this reduction was not significant on day 3. CS = compressive strength, DW = distilled water

Three studies evaluated the CS of CaCl_2_ in a static loading condition [3, 13, 32]. Oloomi et al. [32] evaluated CS of Root MTA (RMTA; Salamifar, Tehran, Iran) mixed with distilled water and RMTA added with 5% CaCl_2_ (RMTA-C). The authors reported that after 3 h, the CS of RMTA specimens was significantly lower than those of RMTA-C group. However, the difference was not significant at longer time intervals (24 h and one week). Subgroup meta-analysis was performed for the other two studies [3, 13] and it was shown that addition of 10% CaCl_2_ to MTA resulted in a decrease in CS on days 1 and 7 compared to MTA mixed with distilled water. However, this reduction was not significant on day 3. In general, regardless of time and percentage of the additive, CaCl_2_ addition resulted in a reduction of CS (Fig. 5).

#### Effect on ST

**Fig. 6:**
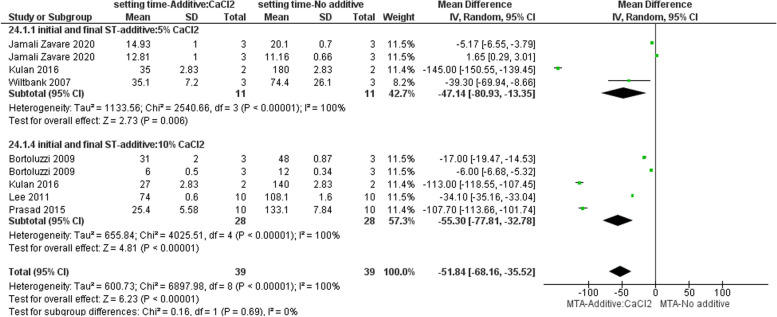
Forest plot of the meta-analysis evaluating the effect of CaCl2 on ST. The addition of both 5% and 10% CaCl2 to white MTA resulted in a reduction in the initial and the final ST. ST = setting time

Seven studies evaluated the ST of MTA with the addition of different proportions of CaCl_2_ [3, 5, 9, 13, 23, 29, 37]. Based on the subgroup meta-analysis, incorporation of both 5% and 10% CaCl2 into white MTA led to a decrease in both initial and final ST (Fig. 6).

#### Effect on pH

**Fig. 7:**
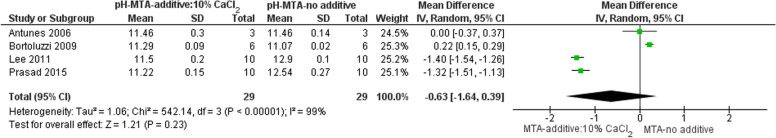
Forest plot of the meta-analysis evaluating the effect of CaCl2 on pH. The addition of 10% CaCl2 has no significant effect on the pH value after 24 h of evaluation

Five studies evaluated pH of MTA following addition of 5% and 10% of CaCl_2_ [3, 5, 9, 13, 36]. Wiltbank et al. compared addition of 5% CaCl_2_ to different brands of MTA and concluded that no significant pH changes could be observed in comparison with control [5]. The meta-analysis indicated that addition of 10% CaCl_2_ has no significant effect on the pH value of the cement after 24 h of evaluation (Fig. 7).

#### Effect on Ca^2+^ ion release

Four studies assessed the release of Ca2 + ions from MTA after the addition of CaCl2. Among these, three studies utilized atomic absorption spectrophotometry, while one study measured calcium hydroxide reaction product formation through decomposition enthalpy as determined by differential scanning calorimetry [23, 30, 33, 36]. It was shown that this addition could increase Ca^2+^ ion release in both 5% and 10% concentrations.

Because of the heterogeneity in data, stemming from variations in time points and measurement methods, a meta-analysis could not be conducted.

#### Effect on cytotoxicity

**Fig. 8:**
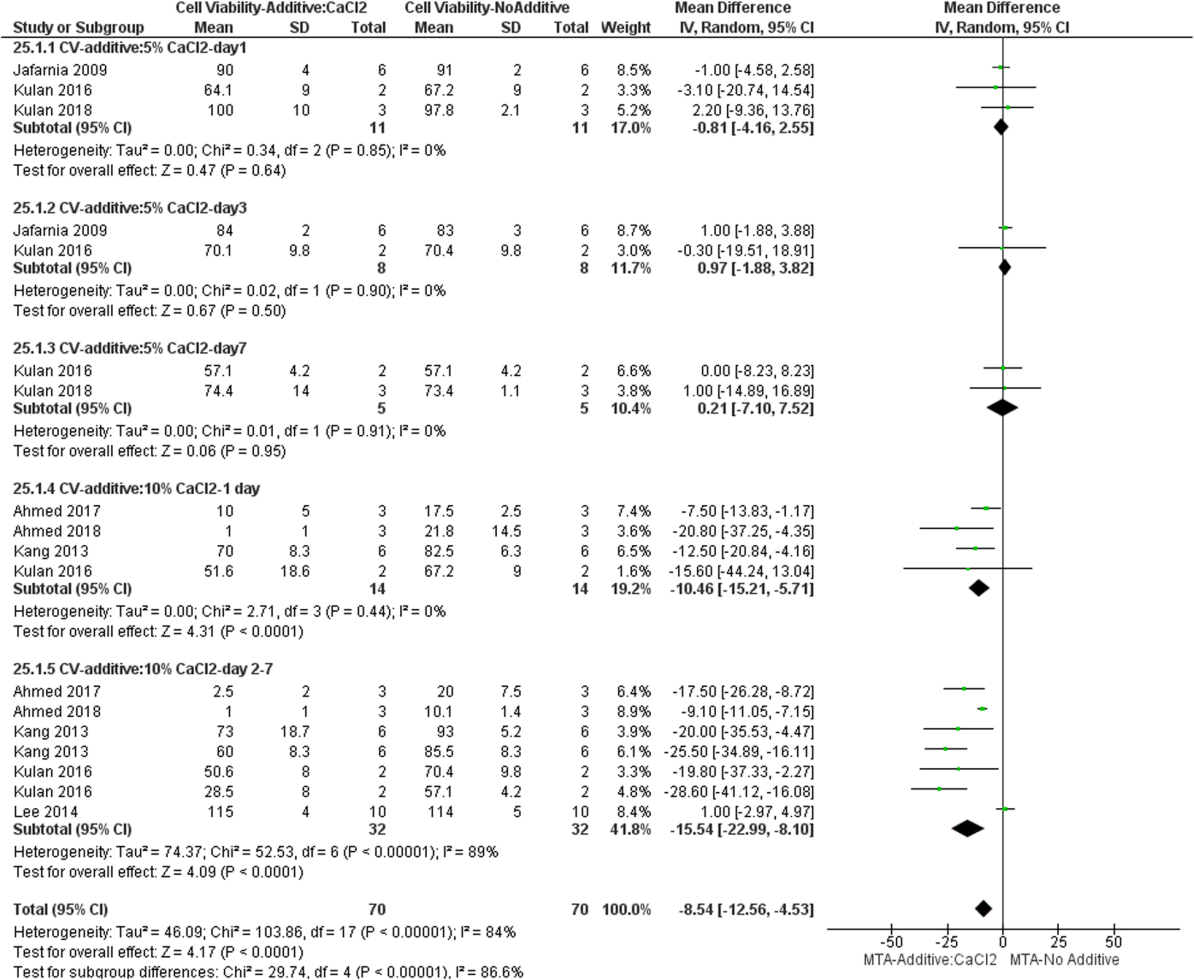
Forest plot of the meta-analysis evaluating the effect of CaCl2 on cytotoxicity. The result of the subgroup meta-analysis indicated that the addition of 5% CaCl2 did not increase cell cytotoxicity of MTA on days 1, 3, and 7. However, the addition of 10% CaCl2 resulted in a significant reduction of cell viability on days 1 to 7 compared to MTA mixed with DW. CV = cell viability

Seven studies evaluated cytotoxicity of MTA following incorporation of different CaCl_2_ concentrations in vitro [14, 26, 27, 29, 31, 33, 34]. The result of the subgroup meta-analysis indicated that addition of 5% CaCl_2_ did not increase cytotoxicity of MTA on days 1, 3, and 7. However, the addition of 10% CaCl_2_ resulted in a significant reduction of cell viability on days 1 to 7 compared to unaltered MTA preparation. Totally, addition of CaCl_2_ resulted in a reduced cell viability compared to control (Fig. 8).

##### Quality assessment of included studies

This systematic review identified 20 studies with a moderate, one study with a high, and four studies with a low risk of bias. The predominant source of bias was noted in the domain of sample size calculation (Table 3).

**Table 3:** Risk of bias assessment of the included studies. Clear description of the parameter = 0. Insufficient or unclear reported parameter = 1, no information of the parameter = 2. A total score between 0 and 3 = low risk of bias, a score of 4 to 7 = moderate-risk, scores of 8 to 10 = high-risk

Author/Year	Samples obtained through a standardized process	Single operator of the machine	Sample size calculation	Blinding of the testing machine operator	Specimens, tests, and formulas according to standard specifications	Total scores	Risk of bias
**Jamali Zavare, F. 2020** [23]	0	1	2	2	0	5	moderate
**Mokhtari, H. 2018** [25]	0	1	1	2	2	6	moderate
**Mokhtari, H. 2018** [24]	0	1	1	2	2	6	moderate
**Kulan, P. 2018** [26]	0	1	2	1	2	6	moderate
**Ahmed, H. 2018** [14]	0	1	2	1	2	6	moderate
**Ahmad, A. 2017** [38]	1	1	2	1	2	7	moderate
**Sobhnamayan, F. 2017** [39]	0	1	1	0	0	2	low
**Marciano, M. A. 2016** [6]	0	1	2	1	0	4	moderate
**Kulan, P. 2016** [29]	0	1	2	1	2	6	moderate
**Ghasemi, N. 2016** [10]	0	1	1	0	0	2	low
**Zapf, A. M. 2015** [30]	0	1	1	2	0	4	moderate
**Prasad, A. 2015** [13]	0	1	1	2	1	5	moderate
**Natu, V. P. 2015** [11]	0	1	2	1	1	5	moderate
**Lee, B. N. 2014** [31]	1	1	2	1	1	6	moderate
**Oloomi, K. 2013** [32]	0	1	1	1	0	3	low
**Kang, J. Y 2013** [33]	0	1	2	2	2	7	moderate
**Duarte, M. A. 2012** [8]	0	1	2	1	0	4	moderate
**Lee, B. N. 2011** [3]	0	1	1	1	0	3	low
**Jafarnia, B. 2009** [34]	0	1	1	2	1	5	moderate
**Bortoluzzi, E. A. 2009** [9]	0	1	2	2	0	5	moderate
**Huang, T. H. 2008** [15]	1	1	2	2	0	6	moderate
**Ding, S. J. 2008** [35]	2	1	2	2	2	9	high
**Wiltbank, K. B. 2007** [5]	1	1	2	1	0	5	moderate
**Antunes Bortoluzzi, E. 2006** [36]	0	1	2	1	0	4	moderate

## Discussion

The result of this meta-analysis revealed that the addition of PG increased the ST of MTA. This increase may result from chemical interaction between the PG and hydrating silicates and aluminate, which yields an amorphous materials in the cement matrix [40]. CS is an indicator of setting reaction. Since moisture is required for the setting reaction of MTA, addition of PG performs a fluidizing action on MTA-water mixture. Thus, a lower amount of water is required to reach a clinically acceptable consistency of MTA. Additionally, PG is a hygroscopic (water-absorbing) compound which can be added to the MTA powder. A reduction in water/powder ratio and water available for powder may decrease the CS of the cement [10, 40]. The results of the included studies were contradictory [10, 39], which can be due to the different experimental settings in the studies. Use of paraffin in the mold-sample interface probably to facilitate separation of the sample from the mold in one study [10], can be the culprit for lower CS after the addition of PG. Another difference between the two studies is the use of different liquids to keep the samples moist during the setting process (distilled water and phosphate-buffered saline). The measured CS of calcium silicate cements is directly influenced by the methodology of evaluation. In various studies, CS is measured after storage of samples in 100% humidity or complete immersion in an aqueous environment. The results of the study by Ha et al. [41] indicated that immersion of the sample into an aqueous environment results in higher CS values compared with those stored in high humidity. Since none of the included studies in this systematic review used water-immersed samples, lower CS values were reported compared with other similar investigations [41, 42].

Hydration products of MTA consist of calcium silicate hydrate (CSH) gel and calcium hydroxide. Ca^2+^ is produced in high proportions from calcium hydroxide and by the decomposition of CSH, which leads to an alkaline pH [43]. The addition of PG results in slower ST, and as a result, Ca^2+^ formation can be sustained for a more extended period which is in accordance with the studies included in this review [6, 8, 11]. If a high PG to water ratio is added to MTA, the water required for completing hydration reaction will be insufficient thereby minimizing formation of hydration products [11]. The Food and Drug Administration (FDA) has classified PG as an additive that is "generally recognized as safe" [44]. Two animal studies revealed that the addition of PG does not increase tissue inflammation more than the amount caused by MTA alone [6, 45].

Disodium hydrogen phosphate might increase the CS of MTA because of its pH. However, this was not in accordance with two similar original investigations [24, 32]. This solution can accelerate hydroxyapatite formation by providing phosphate [46]. Hydroxyapatite can promote cell adhesion and differentiation. Lotfi et al. evaluated inflammatory reactions following subcutaneous implantation of WMTA mixed with disodium hydrogen phosphate in an animal model [47]. The results revealed that addition of this substance reduced inflammatory reactions, but the difference between original and modified WMTA preparations was not significant. This finding was in accordance with the result of the current meta-analysis indicating that addition of 2.5% wt. Na_2_HPO_4_ to MTA does not adversely affect the biocompatibility of the material and might be able to promote further osteo/dentinogenic differentiation.

The result of this meta-analysis showed that addition of both 5% and 10% CaCl_2_ to white MTA resulted in a reduction in both initial and final ST. Acceleration in setting corresponds with more formation of hydration products from the initial stages of setting reaction. This results in a decreased total porosity of admixture, leading to an increase of CS [12]. However, this was not in accordance with the studies included in this systematic review. This controversy may be due to the difference in the percentage of CaCl_2_ added to MTA.

Addition of CaCl_2_ can increase Ca^2+^ ion release and pH mainly because it provides additional Ca^2+^ ions for the cement [36]. However, the result of this meta-analysis indicates that this additive does not significantly alter the pH and Ca^2+^ release of MTA. This might be due to the different laboratory settings in the studies included. Unfortunately, there is no standard specification regarding the dimentional size of the specimens used to evaluate Ca^2+^ ion release and pH. Ca^2+^ ions may be responsible for biological effects of MTA [48]. Therefore, because CaCl_2_ promotes Ca^2+^ ion release of the cement, it is expected that this admixture promotes cell viability and osteo/dentinogenic differentiation. McNamara et al. evaluated biocompatibility of 5% CaCl_2_ added to MTA in an animal model [49]. The authors stated that at the 8th week, the inflammatory reaction of the CaCl_2_ group was not statistically different from the control. The results of the present meta-analysis indicate that cell viability is not significantly affected by the addition of 5% CaCl_2_ to MTA. However, adding 10% CaCl_2_ reduces cell viability. It is possible that the increased quantity of chloride ions may adversely impact the biocompatibility of the cement [50]. Thus, lower percentages of CaCl_2_ are recommended regarding biocompatibility of the cement. All studies included in this systematic review and meta-analysis evaluated cell biocompatibility via tetrazolium salts (MTT and XTT). These evaluations quantitatively assess mitochondrial activity, and the outcomes serve as a benchmark for cellular viability subsequent to exposure to test materials [51]. As stated previously, Ca^2+^ ions released from MTA can lead to odonto/osteogenic differentiation of stem cells, thereby resulting in the production of mineralized materials. In this regard, additional metabolic energy is supplied by enhanced mitochondrial activity as a prerequisite, which, on the other hand, can be misinterpreted as increased cellular proliferation when the cell viability is assessed using MTT or MTS assays. Therefore, it is recommended that viability assays other than use of tetrazolium salts be used to evaluate the cytotoxicity of substances that increase mitochondrial activity. It is important to notice that the findings of this study are applicable solely to the specific brands examined, and combining different additives may yield disparate results due to potential interactions among various materials.

## Conclusion

Addition of 20% PG, 2.5% wt. Na_2_HPO_4_ and 5% CaCl2 to MTA are recommended to improve the admixture's physical, chemical, and cytotoxic properties.

## Data Availability

The data that support the findings of this study are available from the corresponding author upon reasonable request.

## Abbreviations

MTA: Mineral tioxide aggregate
CSC: Calcium silicate-based cement
CS: Compressive strength
ST: Setting time
PG: Propylene glycole

## Footnotes

1 3-(4,5-Dimethylthiazol-2-yl)-2,5-Diphenyltetrazolium Bromide and 3-(4,5-dimethylthiazol-2-yl)-5-(3-carboxymethoxyphenyl)-2-(4-sulfophenyl)-2H-tetrazolium assays.
